# Supplementary material for: Tall Pinus luzmariae trees with genes from P. herrerae
Source: PeerJ. 2020 Feb 26;8:e8648. doi: 10.7717/peerj.8648 (PMC7049253; doi:10.7717/peerj.8648)
Supplement: Figure S1 [file peerj-08-8648-s006.pdf]

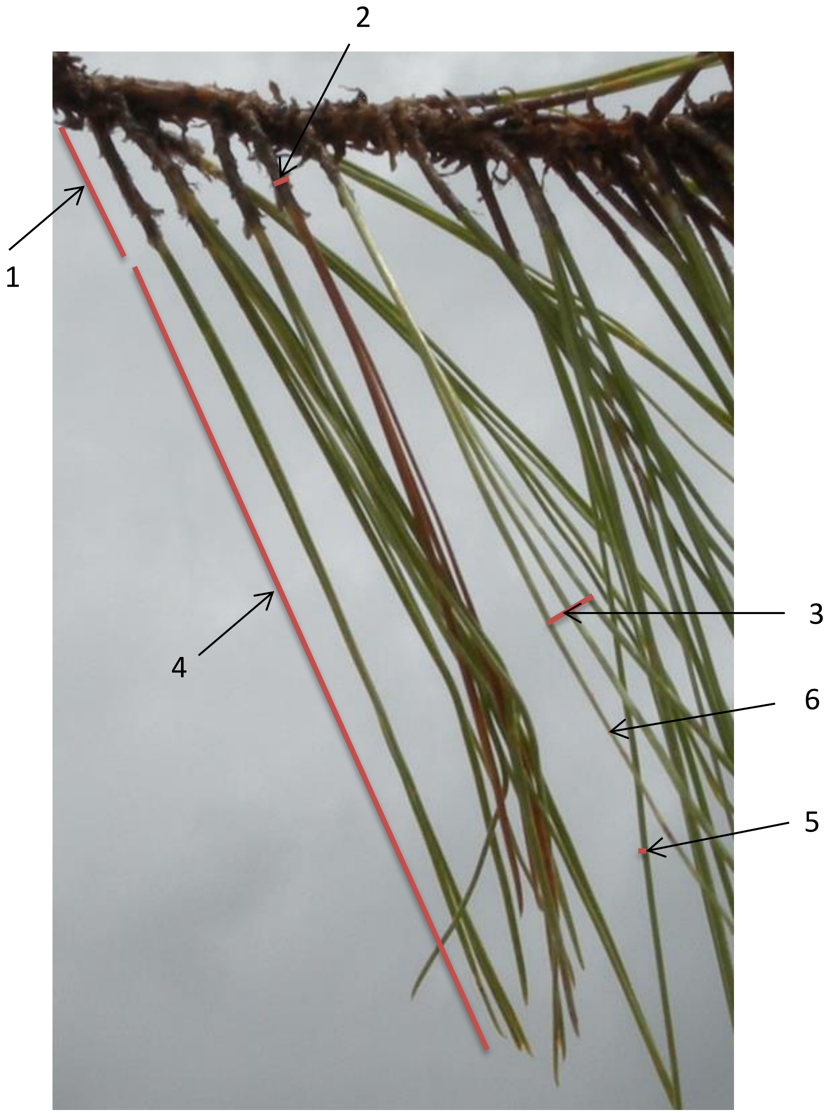

Needle traits

1. Leaf sheath length
2. Leaf sheath diameter
3. Needle number
4. Needle length
5. Needle width
6. Needle thickness

The stomata rows (dorsal face) and stomata rows (ventral faces) are visible only at 40x.

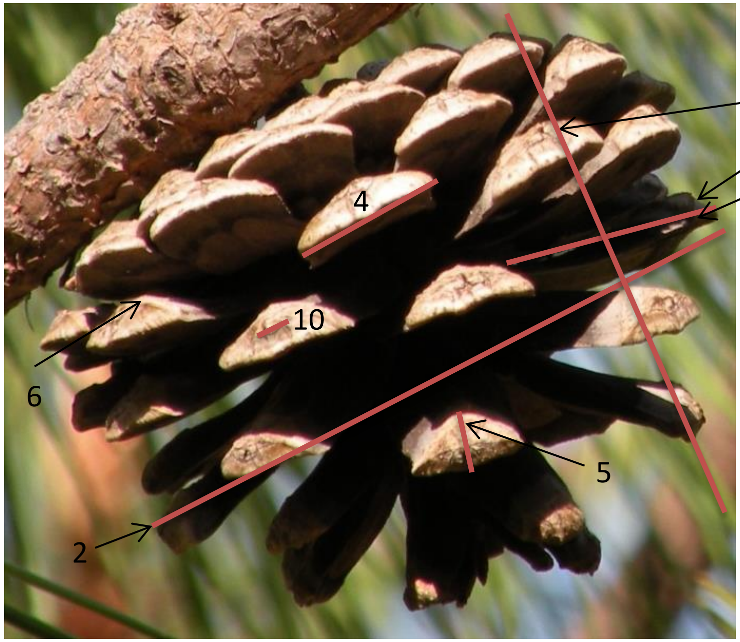

Cone traits

1. Cone width
2. Cone scale position
3. Cone scale length
4. Cone scale width
5. Apophysis width
6. Keel
7. Cone shape (ovoid vs. widely ovoid)
