# Supplementary figures and images for: Tall Pinus luzmariae trees with genes from P. herrerae
Source: PeerJ. 2020 Feb 26;8:e8648. doi: 10.7717/peerj.8648 (PMC7049253; doi:10.7717/peerj.8648)

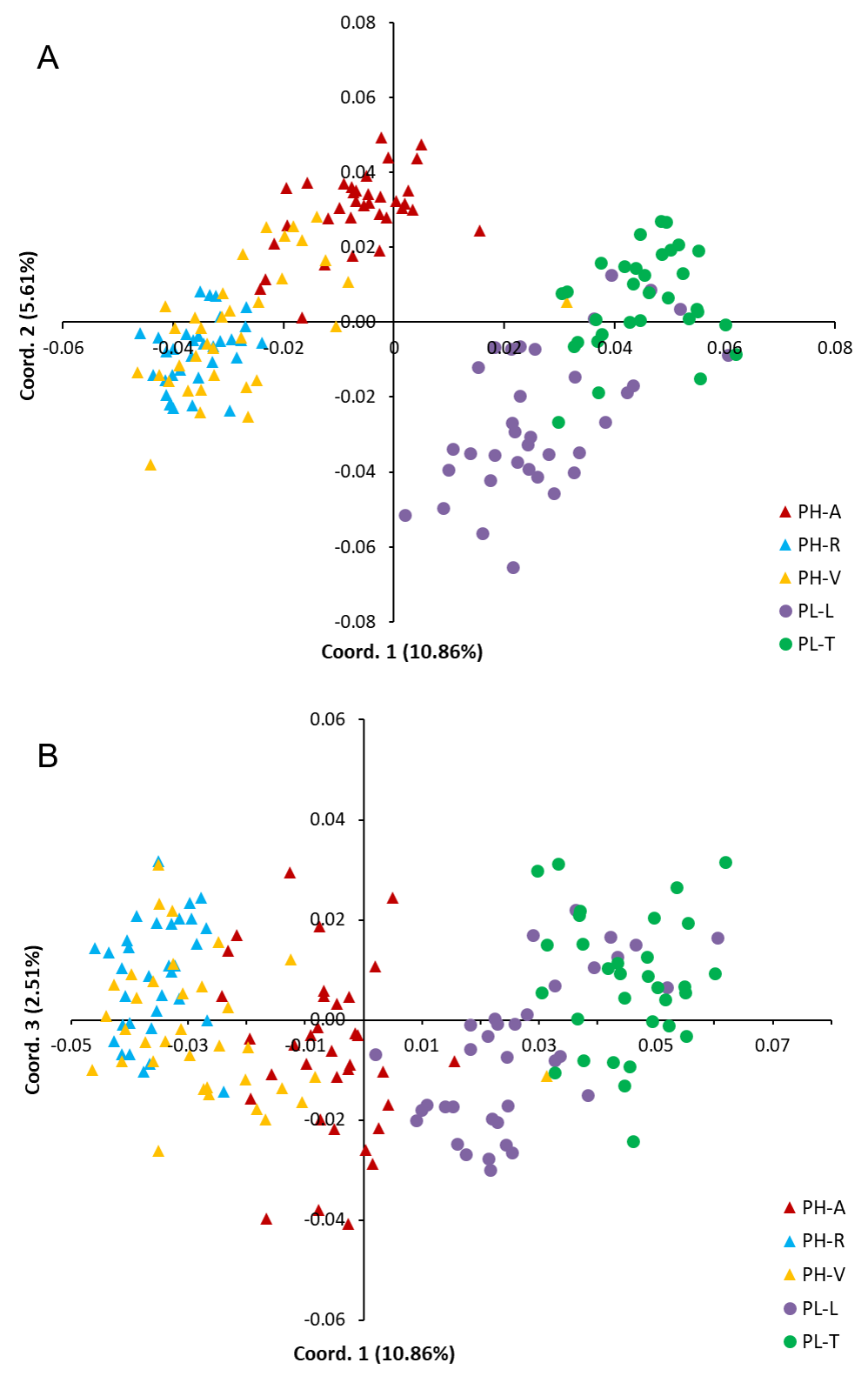

Supplement: Figure S2 — PH-A=Manchon del Abies, PH-R=Ranchito, PH-V=Ventana, PL-L=Laguna and PL-T=Tacuache [file peerj-08-8648-s007.png]

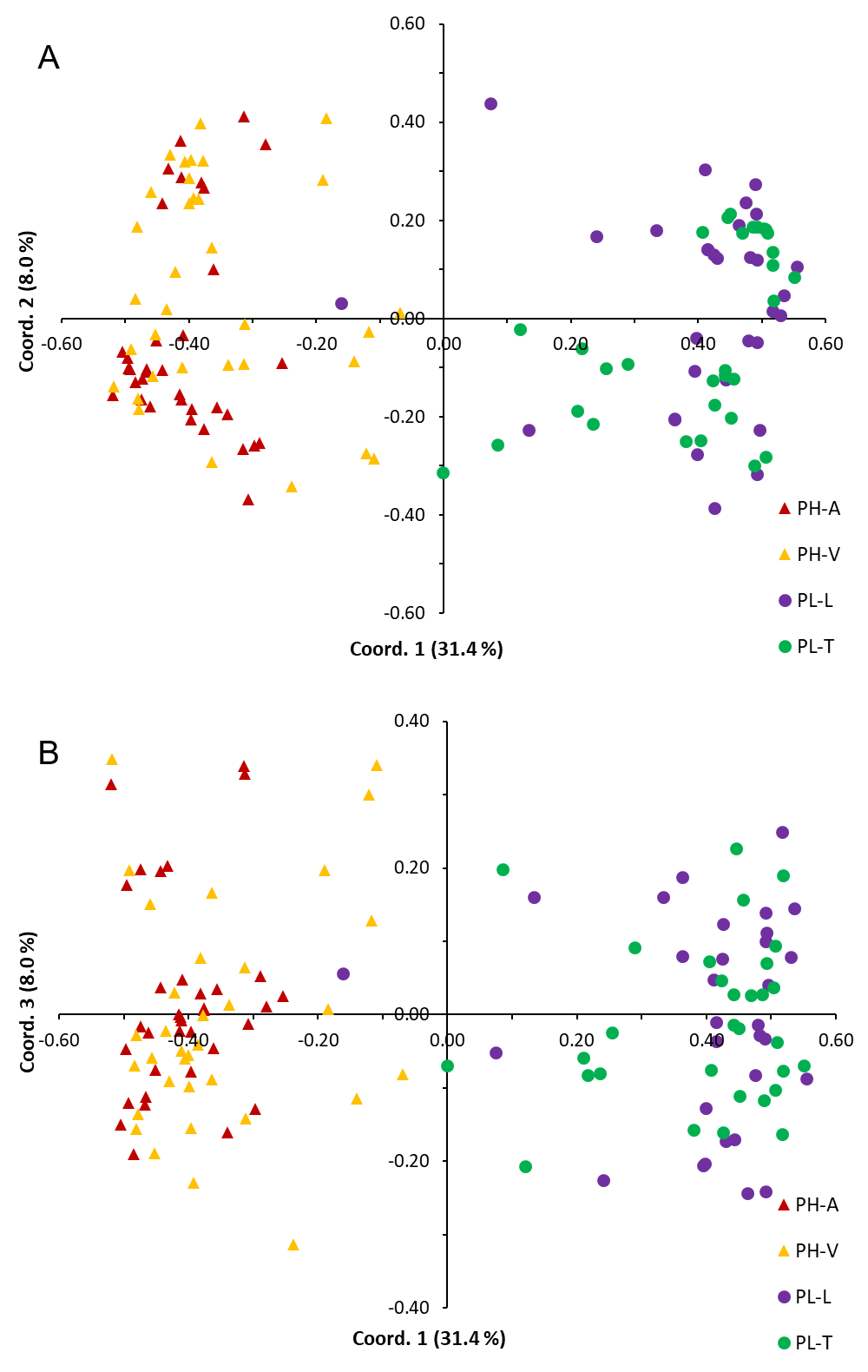

Supplement: Figure S3 — PH-A=Manchon del Abies, PH-V=Ventana, PL-L=Laguna and PL-T=Tacuache [file peerj-08-8648-s008.png]

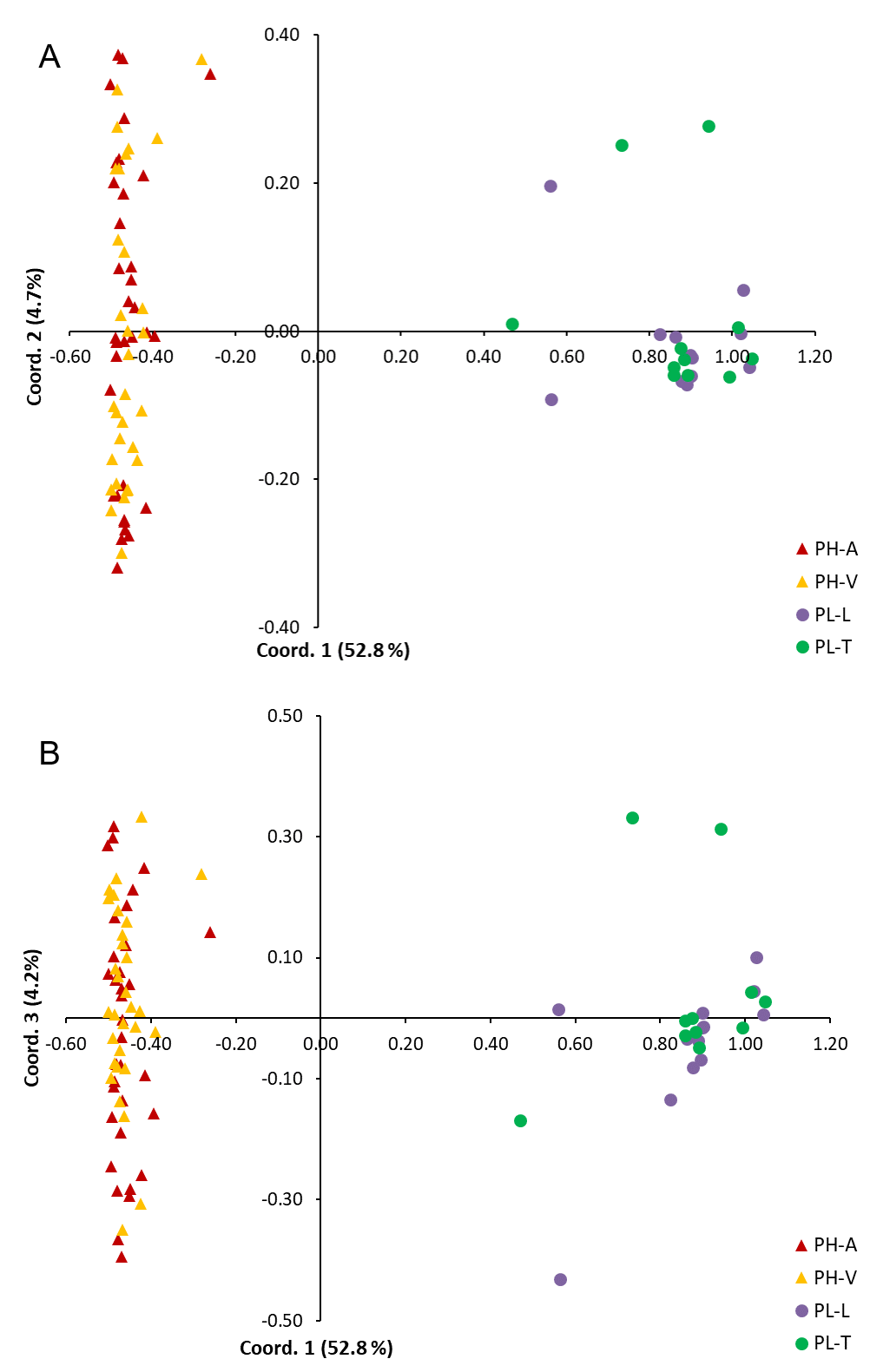

Supplement: Figure S4 — PH-A=Manchon del Abies, PH-V=Ventana, PL-L=Laguna and PL-T=Tacuache. [file peerj-08-8648-s009.png]
